# A new approach for prediction of tumor sensitivity to targeted drugs based on functional data

**DOI:** 10.1186/1471-2105-14-239

**Published:** 2013-07-29

**Authors:** Noah Berlow, Lara E Davis, Emma L Cantor, Bernard Séguin, Charles Keller, Ranadip Pal

**Affiliations:** 1 Department of Electrical and Computer Engineering, Texas Tech University, Lubbock, TX, USA; 2 Department of Pediatrics, Papé Family Pediatric Research Institute, Oregon Health & Science University, Portland, OR, USA; 3 Flint Animal Cancer Center, Colorado State University, Fort Collins, CO, USA

## Abstract

**Background:**

The success of targeted anti-cancer drugs are frequently hindered by the lack of knowledge of the individual pathway of the patient and the extreme data requirements on the estimation of the personalized genetic network of the patient’s tumor. The prediction of tumor sensitivity to targeted drugs remains a major challenge in the design of optimal therapeutic strategies. The current sensitivity prediction approaches are primarily based on genetic characterizations of the tumor sample. We propose a novel sensitivity prediction approach based on functional perturbation data that incorporates the drug protein interaction information and sensitivities to a training set of drugs with known targets.

**Results:**

We illustrate the high prediction accuracy of our framework on synthetic data generated from the Kyoto Encyclopedia of Genes and Genomes (KEGG) and an experimental dataset of four canine osteosarcoma tumor cultures following application of 60 targeted small-molecule drugs. We achieve a low leave one out cross validation error of <10% for the canine osteosarcoma tumor cultures using a drug screen consisting of 60 targeted drugs.

**Conclusions:**

The proposed framework provides a unique input-output based methodology to model a cancer pathway and predict the effectiveness of targeted anti-cancer drugs. This framework can be developed as a viable approach for personalized cancer therapy.

## Background

In the last decade, a number of drugs targeting specific biologically relevant kinases have been developed that are becoming common in cancer research as a basis for personalized therapy. The idea of treating cancer through inhibition of a specific tyrosine kinase was proven by the discovery that patients with Chronic Myeloid Leukemia can be successfully treated by inhibiting the tyrosine kinase BCR-ABL with the kinase inhibitor Imatinib Mesylate [1]. However, the success rate of any one specific targeted drug for other forms of cancer, such as sarcoma, is limited as the tumors exhibit a wide variety of signaling pathways and are not uniformly dependent on the activity of a specific kinase [2-6].

The numerous aberrations in molecular pathways that can produce cancer is one cause to necessitate the use of drug combinations for treatment of individual cancers. Combination therapy design requires a framework for inference of the individual tumor pathways, prediction of tumor sensitivity to targeted drug(s) and algorithms for selection of the drug combinations under different constraints. The current state of the art in predicting sensitivity to drugs is primarily based on assays measuring gene expression, protein abundance and genetic mutations of tumors; these methods often have low accuracy due to the breadth of available expression data coupled with the absence of information on the functional importance of many genetic mutations. A commonly used method for predicting the success of targeted drugs for a tumor sample is based on the genetic aberrations in the tumor (*e.g.* mutation, amplification). However, the accuracy of prediction of drug sensitivity based on mutation knowledge is limited in many forms of tumors as some of the mutations (or low frequency polymorphisms) may not be functionally important or tumors can develop without the known genetic mutations. Statistical tests have been used in [7] to show that genetic mutations can be predictive of the drug sensitivity in non-small cell lung cancers but the classification rates of these predictors based on individual mutations for the aberrant samples are still low. For specific diseases, some mutations have been able to predict the patients that will not respond to particular therapies: for instance [8] reports a success rate of 87% in predicting non-responders to anti-EGFR monoclonal antibodies using the mutational status of KRAS, BRAF, PIK3CA and PTEN. The prediction of tumor sensitivity to drugs has also been approached as a classification problem using gene expression profiles. In [9], gene expression profiles are used to predict the binarized efficacy of a drug over a cell line with the accuracy of the designed classifiers ranging from 64% to 92%. In [10], a co-expression extrapolation (COXEN) approach is used to predict the binarized drug sensitivity in data points outside the training set with an accuracy of around 75%. In [11], a Random Forest based ensemble approach was used for prediction of drug sensitivity and achieved an *R*^2^ value of 0.39 between the predicted *IC*_50_s and experimental *IC*_50_s. Supervised machine learning approaches using genomic signatures achieved a specificity and sensitivity of higher than 70% for prediction of drug response in [12]. Tumor sensitivity prediction has also been considered as (a) a drug-induced topology alteration [13] using phospho-proteomic signals and prior biological knowledge of a generic pathway and (b) a molecular tumor profile based prediction [7,14].

Most interestingly, in the recent cancer cell line encyclopedia (CCLE) study [15], the authors characterize a large set of cell lines (>900) with numerous associated data measurement sets: gene and protein expression profiles, mutation profiles, methylation data along with the response of around 500 of these cells lines across 24 anti-cancer drugs. One of the goals of the study was to enable predictive modeling of cancer drug sensitivity. For generating predictive models, the authors considered regression based analysis across input features of gene and protein expression profiles, mutation profiles and methylation data. The performance (as measured by Pearson correlation coefficient between predicted and observed sensitivity values) of the predictive models using 10 fold cross validation ranged between 0.1 to 0.8. In particular, the correlation coefficient for prediction of sensitivity using genomic signatures for the drug Erlotinib across >450 cell lines was <0.35. Erlotinib is a commonly used tryosine kinase inhibitor selected primarily as an EGFR inhibitor. However, studies have shown [16] that these targeted drugs often have numerous side targets that can play significant roles in the effectiveness of the inhibitor drugs. The target inhibition profiles of drugs and sensitivity of trainings set of drugs can provide significant information for enhanced prediction of anti-cancer drug sensitivity as we have recently shown [17]. By incorporating the drug-target interaction data and sensitivities of training drugs with genomic signatures, we were able to achieve a correlation coefficient of 0.79 (more than 2 fold increase in correlation coefficient) for prediction of Erlotinib sensitivity using 10 fold cross validation. The result illustrates the fundamental concept of the importance of drug-target interaction and functional data under which we develop the sensitivity prediction method presented in this paper. By developing a framework around the functional and target information extracted from the primary tumor drug screen performed by our collaborators, we seek to develop a cohesive approach to sensitivity prediction and combination therapy design. This necessitates the generation of the tumor pathway structure for individual patients to decide on the target inhibitors for therapy based on the personalized patient pathways.

**Figure 1:**
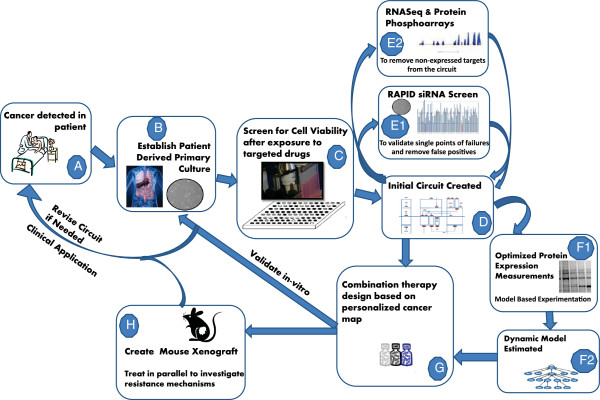
Combination therapy design workflow: various steps in the design of combination targeted therapy.

We envision that the overall schematic of the design of personalized pathways and personalized therapy will be similar to the workflow shown in Figure 1.

The explanations of the various steps in the design process are as follows: 

(Steps A-B) A patient is diagnosed with cancer and a primary culture of the tumor is established.

(Step C) Cell viability after exposure to targeted drugs is measured through a drug screen. Use of this functional data rather than mutation or protein biomarkers provides a unique advantage.

(Step D) A target inhibition map (TIM) is generated based on the *IC*_50_^′^*s* and the known targets of the drugs in the screen. TIM denotes a predictive model that provides the sensitivity for all possible target inhibitions. Specifically, a TIM is composed of a set *T*={*T*_1_,*T*_2_,⋯,*T*_*n*_} consisting of binary variables, each denoting inhibition of a target, and a function *f* relating the target inhibitions to the steady state sensitivity *y*_*T*_, i.e. *y*_*T*_=*f*(*T*_1_,*T*_2_,⋯,*T*_*n*_). The inhibition vector (*e*_1_,*e*_2_,⋯,*e*_*n*_) corresponding to a drug is known as the Drug Target Inhibition Profile (DTIP). A detailed example of TIM is provided in Additional file 1. The coarse structure of the TIM can also be represented by an abstract pathway which will be termed TIM Circuit. The construction of the TIM Circuit is explained in the methods section.

(Steps E1-E2) Further data is collected using siRNA screens [18], RNA sequencing and Protein phosphoarrays to reduce model parameter uncertainties.

(Steps F1-F2) Based on the knowledge of the TIM and TIM-directed protein expression measurements, the dynamic model is created.

(Step G) Combination therapy is designed utilizing the personalized TIM and the dynamic model (if estimated). Various constraints such as avoiding resistance to drugs or minimizing toxicity can be applied to design the combination therapy.

(Step H) A mouse xenograft model [19] can be used to study development of resistance simultaneously.

(Step B revisited) The generated drug combinations are validated *in vitro* on the primary culture.

(Step A revisited) If needed, the circuit is revised or the drug combination with best response *in vivo* (mouse) and *in vitro* (primary culture) is then provided to the patient.

The primary contributions of this paper are: (1) methods for extraction of numerically relevant drug targets from single-run drug screens, (2) design of the personalized TIM circuit based on drug perturbation data, (3) algorithms for sensitivity prediction of a new drug or drug cocktail, (4) validation over canine osteosarcoma primary tumors and (5) pathway flow inference using sequential protein expression measurements. The scope of the present article is concentrated around steps B, C and D of Figure 1.

The perturbation data required for our proposed method originates from a drug screen consisting of 60 small molecule inhibitors with quantified kinase interaction behaviors. This drug screen, denoted Drug Screen Version 1.0, consists of two sets of data: (i) The first set is the experimentally generated drug sensitivities provided as 50% inhibitory concentration (*IC*_50_) values. The *IC*_50_ values denote the amount of a drug required to reduce the population of cancerous cells *in vitro* by half. The sensitivity values are expected to change during each new cell line/ tumor culture experiment. The generation of the sensitivities in step C can be done within 72 hours of initial biopsy using drug sensitivity assays which is a period of limited cell divisions for most primary cultures. Thus, the estimated personalized maps may be *closer to real-time circuits in cancer cells* - akin to the signaling found in an untreated patient within a day or two after biopsy, and not the evolving consensus pattern of signaling for growing and dividing tumor cells as subpopulations emerge with increased fitness *in vitro*. (ii) In addition, the drug screen contains experimentally derived half-maximal concentration (*EC*_50_) values for the interaction of each drug and each kinase target. The *EC*_50_ value is directly related to the notion of inhibition of a kinase target; in particular, the *EC*_50_ values correspond to the amount of a compound needed to deactivate via phosphorylation 50% of the population of the associated target. Hence, for a drug compound, a target with a lower *EC*_50_ is the one that will be heavily inhibited at low drug concentration levels. Thus, low *EC*_50_ targets are often considered to be the primary targets of a drug. The remaining targets are considered to be the side targets of a drug, and are often ignored. The utility of this *EC*_50_ data is its consistency throughout experiments; the *EC*_50_ values as curated from literature searches [16,20] are fixed, regardless of change of tumor type or patient of origin. This provides a great amount of prior information for analysis of the drug screen results, and its usage is supported from the experiments performed in [17].

The overall goal of the methods presented in this paper is to create an input-output mathematical framework for the analysis of and inference on the functional data generated by the drug screens for the purpose of anti-cancer drug sensitivity prediction and inference of personalized tumor survival pathway. The personalized tumor survival pathway refers to the visual circuit diagram generated from the inferred Target Inhibition Map as explained in the methods section. Note that the circuit corresponding to a TIM is only a coarse representation of the TIM for visual understanding of the most probable target combinations whose inhibition can reduce the tumor survival. Since the experiments were conducted on in-vitro cell cultures with the output being cell viability measured in terms of IC50, the survival here refers to tumor cell culture survival and not the overall survival of the patient.

## Results

### TIM Generation for canine osteosarcoma tumor cultures and cross-validation estimates of prediction accuracy

The sensitivity prediction and circuit analysis performed on actual biological data are validations of the proposed methodology to be described in the *Methods* section. The experimental data on four tumor cultures and 60 targeted drug screen panel were generated in the Keller laboratory at OHSU.

The cell lines applied to the drug screen were four canine osteosarcoma cell lines cultured from four distinct canines, denoted Bailey, Charley, Sy, and Cora. The tumor cultures were collected by Dr. Bernard Seguin of Oregon State University from canines that are part of an ongoing clinical trial for osteosarcoma (OSU IACUC approval numbers for this study are 4217 and 4273). The tumor samples were collected from client-owned animals that have developed the disease naturally. All procedures performed on these animals with regards to tumor collection were strictly for treatment purposes and nothing was done different because of the drug perturbation study. All procedures were performed according to standard of care regardless of whether an animal had its tumor sampled.

For the generation of the experimental data, the canine osteosarcoma primary cell cultures were plated in 384 well plates at a seeding density of 2000 cells per well over graded concentrations of 60 small-molecule kinase inhibitors. Each inhibitor was plated individually at four concentrations predicted to bracket the *IC*_50_ for that drug. Cells were cultured in RPMI 1640 supplemented with 2mM glutamine, 2mM sodium pyruvate, 2mM HEPES, 1% penicillin streptomycin, and 10% fetal bovine serum for 72 hours. At the end of the 72 hour incubation, cell viability was assessed using the MTS assay. All values were normalized to the mean of seven wells on each plate containing no drug. The *IC*_50_ for each drug was then determined by identification of the two concentrations bracketing 50% cell viability and application of the following formula: [((*A*−50)/(*A*−*B*))∗(*D*_*B*_−*D*_*A*_)]+*D*_*A*_ where cell viability value above 50% = A (drug dose for this value is *D*_*A*_) and cell viability value below 50% = B (drug dose for this value is *D*_*B*_). The experimentally generated *IC*_50_ values are included as Additional file 2. The experimentally generated sensitivities (in terms of *IC*_50_ values) of the 60 drugs are then scaled to values between 0 and 1.

**Figure 2:**
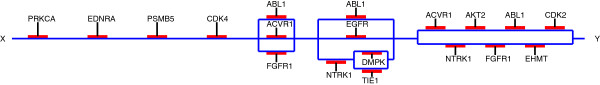
TIM circuit for osteosarcoma primary culture bailey.

**Figure 3:**
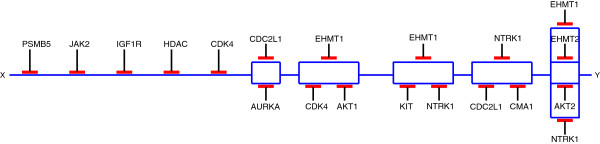
TIM circuit for osteosarcoma primary culture Sy.

Among the 60 drugs on the drug screen, 46 drugs have known target inhibition profiles; of these 46 drugs, 2 provide information only on the target mTOR (mammalian target of Rapamycin) and analysis of these drugs are trivial. Thus, the remaining 44 drugs are used to generate the TIMs. These target profiles were extracted from several literature sources ([16,20]) based on experimental quantitative dissociation constants (*k*_*d*_) which are treated as *EC*_50_ values (explained in the next section) for each drug across kinase target assays with more than 300 targets. The target profiles of the drugs are shown in Additional file 3. Figures 2 and 3 represent the equivalent TIM circuits generated from experimental data for Bailey and Sy respectively. The TIM circuits for Charley and Cora are included in Additional file 1.

To emphasize the biological relevance provided by the TIM framework employed in the analysis of the biological data, we present a more in-depth analysis of the TIM circuit devised for the canine patient Bailey (shown in Figure 2). The vast majority of human osteosarcomas contain genetic or post-translational abnormalities in one or both of the tumor suppressors p53 [21-23] and pRb [24]. The first target identified in this circuit is PKC alpha (PRKCA). PKC alpha modifies CDKN1A (p21), which is the primary mediator of p53 tumor suppressor activity [25]. PSMB5 represents the proteasome (specifically the beta 5 subunit). Previous studies [26] and early preclinical data from the Keller laboratory confirms in vitro sensitivity of many osteosarcomas to proteasome inhibitors and this sensitivity is hypothesized to be due to the integral role of the proteasome in p53 regulation [27]. Interestingly, CDK4 is also prominent in this circuit, which is a primary inhibitor of the tumor suppressor pRb, which is also frequently abnormal in spontaneous human osteosarcoma [24]. CDK2 is an important modifier of both p53 and pRb and is also represented in this circuit [28]. The importance of PI3K pathway in osteosarcoma has also been recently reported using high throughput genotyping [29]. Our TIM circuit includes AKT2 which is downstream of PI3K [30]. Also, EDNRA selected in the circuit has been known to interact with PKC and activate ERK signaling [31].

If the circuit models shown in Figures 2 and 3 are used to predict sensitivities for comparison with experimentally generated data, we will get optimistic results as the models are trained using the entirety of the available data. Thus, we utilize *Leave One Out (LOO)* and 10-fold Cross Validation (10-fold CV) approaches to test the validity of the TIM framework that we present in this paper. For the LOO approach, a single drug among the 44 drugs with known inhibition profiles is removed from the dataset and a TIM is built, using the SFFS suboptimal search algorithm, from the remaining drugs. The resulting TIM is then used to predict the sensitivity of the withheld drug. The predicted sensitivity value is then compared to it’s experimental value; the LOO error for each drug is the absolute value of the experimental sensitivity *y* minus the predicted sensitivity (*y*^′^|*TIM*), i.e. |*y*−(*y*^′^|*TIM*)|. The closer the predicted value is to the experimentally generated sensitivity, the lower the error for the withheld drug. Tables 1, 2, 3 and 4 provides the complete LOO error tables and the average LOO error (Mean Absolute Error: MAE) for each primary culture. The average LOO error over the 4 cell cultures is 0.045 or 4.5%. For the 10-fold cross validation error estimate, we divided the available drugs into 10 random sets of similar size and the testing is done on each fold while being trained on the remaining 9 folds. This is repeated 10 times and average error calculated on the testing samples. We again repeated this experiment 5 times and the average of those mean absolute errors for the primary cell cultures are shown in Table 5. The detailed results of the 10-fold cross validation error analysis are included in Additional file 4. We note that both 10-fold CV and LOO estimates for all the cultures have errors less than 9%, which is extremely low, especially considering the still experimental nature of the drug screening process performed in the Keller laboratory and the available response of only 44 drugs with known target inhibition profile.

**Table 1:** Leave one out error table for osteosarcoma primary culture bailey

**Avg Err**	**Drug**	**Error**	**Pred. Sens**	**Exp. Sens**
0.047	Veliparib (ABT-888)	0.00	0.00	0.00
	Selumetinib (AZD6244)	0.00	0.00	0.00
	Bortezomib	0.00	0.00	0.00
	Bosutinib (SKI-606)	0.02	0.27	0.29
	Dasatinib	0.00	0.91	0.91
	Erlotinib	0.00	0.00	0.00
	Panobinostat (LBH-589)	0.07	0.93	1.00
	Pazopanib (GW-786034)	0.00	0.00	0.00
	PI-103	0.00	0.00	0.00
	Sorafenib	0.00	0.00	0.00
	Vorinostat (SAHA)	0.08	0.93	0.85
	Obatoclax (GX15-070)	0.01	0.28	0.27
	Crizotinib (PF-2341066)	0.00	0.48	0.48
	MK-2206	0.00	0.65	0.65
	Vismodegib (GDC-0449)	0.00	0.00	0.00
	Alisertib (MLN8237)	0.00	0.00	0.00
	SNS-032 (BMS-387032)	0.03	0.66	0.69
	Carfilzomib	0.36	0.64	1.00
	Imatinib	0.02	0.21	0.19
	BIX 01294	0.18	0.65	0.83
	BMS-754807	0.00	1.00	1.00
	SJ-172550	0.00	0.00	0.00
	Barasertib (AZD1152-HQPA)	0.00	0.00	0.00
	Ruxolitinib (INCB018424)	0.00	0.00	0.00
	Cediranib (AZD2171)	0.03	0.45	0.48
	Lapatinib	0.00	0.00	0.00
	Sunitinib	0.01	0.19	0.20
	Trichostatin A	0.06	0.93	0.86
	Tozasertib (VX-680)	0.02	0.58	0.60
	Enzastaurin	0.36	0.64	1.00
	PD0332991	0.36	0.64	1.00
	Valproate	0.00	0.00	0.00
	Resveratrol	0.00	0.00	0.00
	Zibotentan (ZD4054)	0.36	0.64	1.00
	SP600125	0.00	0.00	0.00
	Ponatinib (AP24534)	0.00	0.00	0.00
	BIX 02188	0.00	0.00	0.00
	RO4929097	0.00	0.00	0.00
	Curcumin	0.00	1.00	1.00
	Sodium butyrate	0.00	0.00	0.00
	GANT61	0.11	0.58	0.47
	Aurothiomalate	0.00	0.00	0.00
	(OSI-906)	0.00	0.00	0.00
	Pelitinib (EKB-569)	0.03	0.91	0.88

Pred. Sens. = Predicted Sensitivity, Exp. Sens. = Experimental Sensitivity, Error = |*Pred. Sens.* −*Exp. Sens.*|, Avg Err = Average of the 44 leave one out Errors.

**Table 2:** Leave one out error table for osteosarcoma primary culture charley

**Avg Err**	**Drug**	**Error**	**Pred. Sens**	**Exp. Sens**
0.040	Veliparib (ABT-888)	0.00	0.00	0.00
	Selumetinib (AZD6244)	0.00	0.00	0.00
	Bortezomib	0.00	0.00	0.00
	Bosutinib (SKI-606)	0.00	0.00	0.00
	Dasatinib	0.02	0.94	0.96
	Erlotinib	0.00	0.00	0.00
	Panobinostat (LBH-589)	0.00	1.00	1.00
	Pazopanib (GW-786034)	0.00	0.00	0.00
	PI-103	0.00	0.00	0.00
	Sorafenib	0.00	0.00	0.00
	Vorinostat (SAHA)	0.21	1.00	0.79
	Obatoclax (GX15-070)	0.00	0.30	0.30
	Crizotinib (PF-2341066)	0.01	0.64	0.63
	MK-2206	0.03	0.61	0.65
	Vismodegib (GDC-0449)	0.00	0.00	0.00
	Alisertib (MLN8237)	0.00	1.00	1.00
	SNS-032 (BMS-387032)	0.00	1.00	1.00
	Carfilzomib	0.33	0.67	1.00
	Imatinib	0.00	0.00	0.00
	BIX 01294	0.18	0.68	0.86
	BMS-754807	0.00	1.00	1.00
	SJ-172550	0.00	0.00	0.00
	Barasertib (AZD1152-HQPA)	0.00	0.00	0.00
	Ruxolitinib (INCB018424)	0.00	0.00	0.00
	Cediranib (AZD2171)	0.02	0.43	0.44
	Lapatinib	0.00	0.00	0.00
	Sunitinib	0.05	0.88	0.82
	Trichostatin A	0.00	1.00	1.00
	Tozasertib (VX-680)	0.00	1.00	1.00
	Enzastaurin	0.00	0.00	0.00
	PD0332991	0.00	0.00	0.00
	Valproate	0.00	0.00	0.00
	Resveratrol	0.00	0.00	0.00
	Zibotentan (ZD4054)	0.33	0.67	1.00
	SP600125	0.00	0.00	0.00
	Ponatinib (AP24534)	0.00	0.00	0.00
	BIX 02188	0.00	1.00	1.00
	RO4929097	0.00	0.00	0.00
	Curcumin	0.00	1.00	1.00
	Sodium butyrate	0.00	0.00	0.00
	GANT61	0.57	0.43	1.00
	Aurothiomalate	0.00	0.00	0.00
	(OSI-906)	0.00	1.00	1.00
	Pelitinib (EKB-569)	0.00	1.00	1.00

Pred. Sens. = Predicted Sensitivity, Exp. Sens. = Experimental Sensitivity, Error = |*Pred. Sens.* −*Exp. Sens.*|, Avg Err = Average of the 44 leave one out Errors.

**Table 3:** Leave one out error table for osteosarcoma primary culture cora

**Avg Err**	**Drug**	**Error**	**Pred. Sens**	**Exp. Sens**
0.036	Veliparib (ABT-888)	0.00	0.00	0.00
	Selumetinib (AZD6244)	0.00	0.00	0.00
	Bortezomib	0.01	1.00	0.99
	Bosutinib (SKI-606)	0.00	0.00	0.00
	Dasatinib	0.03	0.83	0.85
	Erlotinib	0.00	0.00	0.00
	Panobinostat (LBH-589)	0.05	0.96	1.00
	Pazopanib (GW-786034)	0.00	0.00	0.00
	PI-103	0.00	0.00	0.00
	Sorafenib	0.00	0.00	0.00
	Vorinostat (SAHA)	0.13	0.91	0.78
	Obatoclax (GX15-070)	0.01	0.42	0.44
	Crizotinib (PF-2341066)	0.04	0.66	0.69
	MK-2206	0.28	0.66	0.93
	Vismodegib (GDC-0449)	0.00	0.00	0.00
	Alisertib (MLN8237)	0.00	0.00	0.00
	SNS-032 (BMS-387032)	0.00	1.00	1.00
	Carfilzomib	0.01	0.99	1.00
	Imatinib	0.00	0.00	0.00
	BIX 01294	0.23	1.00	0.89
	BMS-754807	0.00	1.00	1.00
	SJ-172550	0.00	0.00	0.00
	Barasertib (AZD1152-HQPA)	0.00	0.00	0.00
	Ruxolitinib (INCB018424)	0.00	0.00	0.00
	Cediranib (AZD2171)	0.31	0.44	0.75
	Lapatinib	0.00	0.00	0.00
	Sunitinib	0.02	0.78	0.76
	Trichostatin A	0.05	0.89	0.96
	Tozasertib (VX-680)	0.00	1.00	1.00
	Enzastaurin	0.00	0.00	0.00
	PD0332991	0.24	0.76	1.00
	Valproate	0.00	0.00	0.00
	Resveratrol	0.00	0.00	0.00
	Zibotentan (ZD4054)	0.00	0.00	0.00
	SP600125	0.00	0.00	0.00
	Ponatinib (AP24534)	0.00	0.00	0.00
	BIX 02188	0.03	0.92	0.89
	RO4929097	0.00	0.00	0.00
	Curcumin	0.00	0.00	0.00
	Sodium butyrate	0.00	0.00	0.00
	GANT61	0.00	0.00	0.00
	Aurothiomalate	0.00	0.00	0.00
	(OSI-906)	0.11	1.00	0.89
	Pelitinib (EKB-569)	0.01	0.62	0.63

Pred. Sens. = Predicted Sensitivity, Exp. Sens. = Experimental Sensitivity, Error = |*Pred. Sens.* −*Exp. Sens.*|, Avg Err = Average of the 44 leave one out Errors.

**Table 4:** Leave one out error table for osteosarcoma primary culture Sy

**Avg Err**	**Drug**	**Error**	**Pred. Sens**	**Exp. Sens**
0.056	Veliparib (ABT-888)	0.00	0.00	0.00
	Selumetinib (AZD6244)	0.00	0.00	0.00
	Bortezomib	0.00	0.00	0.00
	Bosutinib (SKI-606)	0.00	0.59	0.59
	Dasatinib	0.01	0.63	0.62
	Erlotinib	0.00	0.00	0.00
	Panobinostat (LBH-589)	0.05	0.86	0.80
	Pazopanib (GW-786034)	0.00	0.00	0.00
	PI-103	0.00	0.00	0.00
	Sorafenib	0.00	0.00	0.00
	Vorinostat (SAHA)	0.11	0.60	0.71
	Obatoclax (GX15-070)	0.00	0.00	0.00
	Crizotinib (PF-2341066)	0.00	0.59	0.59
	MK-2206	0.04	0.53	0.58
	Vismodegib (GDC-0449)	0.00	0.00	0.00
	Alisertib (MLN8237)	0.00	0.00	0.00
	SNS-032 (BMS-387032)	0.06	0.62	0.69
	Carfilzomib	0.38	0.62	1.00
	Imatinib	0.00	0.00	0.00
	BIX 01294	0.20	1.00	0.82
	BMS-754807	0.01	0.53	0.54
	SJ-172550	0.00	0.00	0.00
	Barasertib (AZD1152-HQPA)	0.00	0.00	0.00
	Ruxolitinib (INCB018424)	0.00	1.00	1.00
	Cediranib (AZD2171)	0.44	0.30	0.75
	Lapatinib	0.00	0.00	0.00
	Sunitinib	0.02	0.60	0.58
	Trichostatin A	0.20	0.80	1.00
	Tozasertib (VX-680)	0.00	1.00	1.00
	Enzastaurin	0.00	0.00	0.00
	PD0332991	0.05	0.62	0.67
	Valproate	0.00	0.00	0.00
	Resveratrol	0.00	0.00	0.00
	Zibotentan (ZD4054)	0.00	0.00	0.00
	SP600125	0.00	0.00	0.00
	Ponatinib (AP24534)	0.00	0.00	0.00
	BIX 02188	0.00	0.00	0.00
	RO4929097	0.00	0.00	0.00
	Curcumin	0.00	0.00	0.00
	Sodium butyrate	0.00	0.00	0.00
	GANT61	0.38	0.31	0.69
	Aurothiomalate	0.00	0.00	0.00
	(OSI-906)	0.47	0.53	1.00
	Pelitinib (EKB-569)	0.00	0.00	0.00

Pred. Sens. = Predicted Sensitivity, Exp. Sens. = Experimental Sensitivity, Error = |*Pred. Sens.* − Exp. Sens.|, Avg Err = Average of the 44 leave one out Errors.

**Table 5:** Cross validation results

**Cell culture**	**Average MAE**
Bailey	0.080
Charley	0.087
Cora	0.083
Sy	0.072

The 10 fold- cross valdiation error estimates were calculated for 5 runs and the average mean absolute error (MAE) across all the drugs is shown.

To provide a measure of the overlap between drugs, we considered a similarity measure *Λ*(*D*_1_,*D*_2_) based on the *EC*_50_ of the drugs *D*_1_ and *D*_2_. Let the *EC*_50_^′^*s* of the drugs *D*_1_ and *D*_2_ be given by the *n*-length vectors *E*_1_ and *E*_2_ where *n* denotes the number of drug targets. The entries for the targets that are not inhibited by the drugs (i.e. no *EC*_50_ value available) are set to 0. Let the vectors *V*_1_ and *V*_2_ represent the binarized targets of the drugs i.e. it has a value of 1 if the target is inhibited by the drug and a value of zero if the target is not inhibited by the drug. Then, we define the similarity measure *Λ* as:

(1) $$\Lambda (D_{1},D_{2})=\frac{\overset{n}{\underset{i=1}{\sum }}\text{min}(E_{1}(i),E_{2}(i))*V_{1}(i)*V_{2}(i)}{\overset{n}{\underset{i=1}{\sum }}\text{max}(E_{1}(i),E_{2}(i))}$$

Note that *Λ*(*D*_1_,*D*_1_)=1 and similarity between drugs with no overlapping targets is zero. If two drugs have 50% targets overlapping with same *EC*_50_^′^*s*, then the similarity measure is 0.5. The similarities between the drugs are shown in Additional file 5. Note that except two drugs Rapamycin and Temsirolimus that have a similarity measure of 0.989, all other drugs have significantly lower similarities with each other. The maximum similarity between two different drugs (other than Rapamycin and Temsirolimus) is 0.169. This shows that any two drugs in the drug screen are not significantly overlapping and the prediction algorithm is still able to predict the response.

The low error rate illustrates the accuracy and effectiveness of this novel method of modeling and sensitivity prediction. Furthermore, these error rates are significantly lower than those of any other sensitivity prediction methodology we have found. Consistent with the analysis in [17], the sensitivity prediction rates improve dramatically when incorporating more information about drug-protein interaction. To more effectively compare the results generated via the TIM framework with the results in [15], we also present the correlation coefficients between the predicted and experimental drug sensitivity values in Table 6. The correlation coefficients for predicted and experimentally generated sensitivities for 24 drugs and more than 500 cell lines ranges from 0.1 to 0.8 when genomic characterizations are used to predict the drug sensitivities in the CCLE study [15]. In comparison, our approach based on sensitivity data on training set of drugs and drug-protein interaction information produced correlation coefficients >0.92 (Table 6) for both leave one out (LOO) and 10-fold cross validation (10-fold CV) approaches for error estimation.

**Table 6:** Correlation coefficients of predicted sensitivities vs. experimental sensitivities

**Cell line**	***ρ*LOO**	***ρ*10-fold CV**
Bailey	0.97	0.92
Charley	0.97	0.95
Cora	0.98	0.94
Sy	0.94	0.92

*ρ* LOO = Average Pearson Correlation Coefficient for test samples selected based on Leave One Out approach, *ρ* 10-fold CV = Average Pearson Correlation Coefficient for test samples selected based on 10 fold cross validation approach.

It should be noted that the sensitivity prediction is performed in a continuous manner, not discretely, and thus effective dosage levels can be inferred from the predictions made from the TIM. This shows that the TIM framework is capable of predicting the sensitivity to anti-cancer targeted drugs outside the training set, and as such is viable as a basis for a solution to the complicated problem of sensitivity prediction.

In addition, we tested the TIM framework using synthetic data generated from a subsection of a human cancer pathway taken from the KEGG database [32]. Here, the objective is to show that the proposed TIM method generates models that highly represent the underlying biological network which was sampled via synthetic drug perturbation data. This experiment replicates in synthesis the actual biological experiments performed at the Keller laboratory at OHSU. To utilize the TIM algorithm, a panel of 60 targeted drugs pulled from a library of 1000 is used as a training panel to sample the randomly generated network. Additionally, a panel of 40 drugs is drawn from the library to serve as a test panel. The training panel and the testing panel have no drugs in common. Each of the 60 training drugs is applied to the network, and the sensitivity for each drug is recorded. The generated TIM is then sampled using the test panel which determines the predicted sensitivities of the test panel. The synthetic experiments were performed for 40 randomly generated cancer subnetworks for each of *n*=6,⋯,10 active targets in the network. The active targets are those which, when inhibited, may have some effect on the cancer downstream. To more accurately mimic the Boolean nature of the biological networks, a drug which does not satisfy any of the Boolean network equations will have sensitivity 0, a drug which satisfies at least one network equation will have sensitivity 1. The inhibition profile of the test drugs is used to predict the sensitivity (0 or 1) of the new drug. The average number of correctly predicted drugs for each *n* is reported in Table 7. This synthetic modeling approach generally produces respectable levels of accuracy, with accuracies ranging from 89% to 99%. 60 drugs for training mimics the drug screen setup used by our collaborators and testing 20 drugs for predicted sensitivity approximates a secondary drug screen to pinpoint optimal therapies. The performance of the synthetic data shows fairly high reliability of the predictions made by the TIM approach.

**Table 7:** Results of synthetic experiments based on KEGG pathways

**Targets**	**Correct prediction**	**Accuracy percentage**
n = 6	39.83	99.56
n = 7	38.68	96.69
n = 8	38.18	95.44
n = 9	36.80	92.00
n = 10	35.63	89.06

Correct Prediction = Average number of correct prediction for 40 testing drugs for 40 different synthetic pathways.

We have also tested our algorithm on another set of randomly generated synthetic pathways. The detailed results of the experiment are included in Additional file 1. A large number of testing samples were used for each pathway prediction and the results indicate an average error of less than 10% for multiple scenarios. In comparison, the average error with random predictions was 44%. The average correlation coefficient of the prediction to actual sensitivity for the 8 sets of experiments (each including 10 or 25 different pathways) was 0.91. The average correlation coefficient with random predictions was 0. We also report the standard deviation of the errors and for a representative example, the 10 percentile of the error was -0.154 and 90 percentile 0.051, thus the 80% prediction interval for prediction *μ* was [*μ*−0.154 *μ*+0.051].

The results of the synthetic experiments on different randomly generated pathways shows that the approach presented in the paper is able to utilize a small set of training drugs from all possible drugs to generate a high accuracy predictive model.

## Methods

In this section, we provide an overview of the model design and inference from drug perturbation data for personalized therapy.

### Mathematical formulation

Let us consider that we have drug *IC*_50_ data for a new primary tumor after application of *m* drugs in a controlled drug screen. Let the known multi-target inhibiting sets for these drugs be denoted by *S*_1_, *S*_2_,..., *S*_*m*_ obtained from drug inhibition studies [16,20,33].

The elements of set *S*_*i*_ are *e*_*i*_= [*e*_*i*,1_,*e*_*i*,2_,⋯,*e*_*i*,*n*_] for *i*=1,2,⋯,*m*, where *e*_*i*,*j*_ are real-valued elements describing the interaction of *S*_*i*_ with *K*= [*k*_1_,*k*_2_,⋯,*k*_*n*_], the set of all kinase targets included in the drug screen. The *e*_*i*,*j*_’s refer to the *EC*_50_ values discussed previously. It should be noted that for all *S*_*i*_, *e*_*i*,*j*_ will most often be blank or an extremely high number denoting no interaction.

The initial problem we wish to solve is to identify the minimal subset of *K*, the set of all tyrosine kinase targets inhibited by the *m* drugs in the drug panel, which explains numerically the various responses of the *m* drugs. Denote this minimal subset of *K* as *T*. The rationale behind minimization of *T* is twofold. First, as with any classification or prediction problem, a primary goal is avoidance of overfitting. Secondly, by minimizing the cardinality of the target set required to explain the drug sensitivities found in the exploratory drug screen, the targets included have supportable numerical relevance increasing the likelihood of biological relevance. Additional targets may increase the cohesiveness of the biological story of the tumor, but will not have numerical evidence as support. This set *T* will be the basis of our predictive model approach to sensitivity prediction.

Before formulation of the problem for elucidating *T*, let us consider the nature of our desired approach to sensitivity prediction. From the functional data gained from the drug screen, we wish to generate a personalized tumor survival pathway model instead of a linear function approximator with minimal error. We are working under the fundamental assumption that the tumor survival pathway is nonlinear in its behavior; this assumption is reasonable given the difficulty in treating multiple forms of cancer. One frequent theory in personalized therapy is that effective treatment results from applying treatment across multiple important biological pathways. These pathways generally consist of sequentially activated gene and protein nodes acting as a feedback network. Treatment of individual pathways may not be sufficient for majority of diseases, so multiple independent parallel pathways must be targeted to create an effective treatment. We believe that one possible approach to the analysis of multiple pathway treatment is to begin with an underlying framework based on the Boolean interactions of the multiple targets in the pathway architecture. The approach is based on developing families of Boolean equations that describe the multiple treatment combinations capable of acting as an effective intervention strategy. For the initial step of developing the underlying Boolean functions, an initial binarization of the data set must be performed. However, the resulting model lends itself to numerous continuous approaches to sensitivity prediction which we will explore further in the paper.

### Binarization of drug targets and conversion of *IC*_50_^′^*s* to sensitivities

In this subsection, we present algorithms for generation of binarized drug targets (1 denoting that the target is inhibited by the drug and 0 denoting that the target is not modified by the drug) and continuous sensitivity score of each drug (a number between 0 and 1 with a higher value denoting that the drug is effective on the tumor). The inputs for the algorithms in this subsection are the *EC*_50_^′^*s* of the drug targets and the *IC*_50_^′^*s* of the drugs when applied to a tumor culture.

In order to perform the binarization, we must consider the nature of the data we are given. In particular, we are provided with an *IC*_50_ for each drug (the concentration of the drug necessary to eliminate 50% of the tumor cell population), and an *EC*_50_ value for each kinase target inhibited by the drug. Under the assumption that the primary mechanism of tumor eradication is, in fact, the protein kinase inhibition enacted by these targeted drugs, a natural consequence would be the existence of a relationship between the *IC*_50_ and *EC*_50_ values. This relationship is explained as such: suppose for a drug *S*_*i*_ the *IC*_50_ value of *S*_*i*_ and the *EC*_50_ of kinase target *k*_*j*_, (*k*_*j*_·*e*_*i*,*j*_) are of similar value, then it can be reasonably assumed that kinase target *k*_*j*_ is possibly a primary mechanism in the effectiveness of the drug. In other words, if 50% inhibition of a kinase target directly correlates with 50% of the tumor cells losing viability, then inhibition of the kinase target is most likely one of the causes of cell death. Hence, the target that matches the drug *IC*_50_ is binarized as a *target hit* for the drug.

The above assumption of direct correlation for all successful drugs is obviously an extremely restrictive assumption and will be unable to produce high accuracy predictions. Thus, the binarization scheme has to be modified to incorporate the following three factors: 

First: noises in varying magnitude will be present in the drug screen data generated by our collaborators. The noise is unavoidable, and as such, needs to be accounted for. In addition, despite the high accuracy of the drug-protein interaction data procured from literature, we should still account for possible errors in the *EC*_50_ values for the numerous drugs.

Second: the restrictive assumption considers that effective drugs operate on single points of failure within the patient’s signaling pathway. In reality, high sensitivity to a drug is often attributed to a family of related kinases (such as the Aurora kinase family) or several independent kinases working synergistically over one or multiple pathways to induce tumor death. This cooperative multivariate behavior needs to be taken into account while binarizing a drug to its multiple possible targets.

Third: despite the high level of currently available knowledge on the biological effects of numerous targeted drugs, there remains the possibility of a drug having high sensitivity while having no known mechanisms explaining its sensitivity. Therefore, we must consider the situation where there are latent mechanisms not considered within the dataset that are proving to be effective in some combination of treatment. This point does not necessarily eliminate the possibility of kinase mechanisms being an important factor.

We address all three concerns as follows: (1) By considering the log scaled *EC*_50_ values for each target and the log scaled *IC*_50_ value for each drug, we convert the multiplicative noise to additive noise. In addition, we employ scalable bounds around the *IC*_50_^′^*s* to determine binarization values of the numerous kinase targets for each drug. The bounds can be scaled to allow targets that may have *EC*_50_^′^*s* higher than the *IC*_50_ to be considered as a possible treatment mechanism. (2) We extend the bounds to low *EC*_50_ levels, and often down to 0, to incorporate the possibility of target collaboration at various different *EC*_50_ levels. While a high *IC*_50_ indicates the likelihood of drug side targets as therapeutic mechanisms, it does not preclude the possibility of a joint relationship between a high *EC*_50_ target and a low *EC*_50_ target. Hence, to incorporate the numerous possible effective combinations implied by the *IC*_50_ of an effective drug, the binarization range of targets for a drug is the range *α* · log(*IC*_50_)≤ log(*EC*_50_)≤*β* · log(*IC*_50_) where 0≤*α*≪*β*. (3) For reliability and validity of the target set that we aim to construct, it is important to keep *β* in a reasonable range, i.e. *β* should be a smaller constant such as 3 or 4. For the situation where the above bounds do not result in at least one binarized target, the immediate option is to eliminate the drug from the data set before target selection. This prevents incomplete information from affecting the desired target set. As information concerning the drug screen agents gradually becomes complete with respect to other forms of data, such as gene interaction data, additional mechanisms for unexplained targets can be explored and incorporated more readily into the predictive model. With binarization of the data set as explained, we now present the minimization problem that produces a numerically relevant set of targets, *T*.

Consider the target set *T*= [*T*_1_,*T*_2_,⋯,*T*_*n*_], where *T*_*i*_∈{0,1}. Here, 1 denotes inclusion in the target set T and 0 denotes exclusion. For any target set *T*_0_, one can find the representation under *T*_0_ of each drug *S*_*i*_,*i*∈1,⋯,*m* as (*S*_*i*_|*T*_0_) = [*e*_*i*,1_·*T*_1_,*e*_*i*,2_·*T*_2_,⋯,*e*_*i*,*n*_·*T*_*n*_]. As the *T*_0_ will be the basis of the new representation for each drug, this will result in *n*_0_ columns which will be 0 for all *S*_*i*_, where *n*_0_ is the number of *T*_*i*_=0, i.e. the number of targets not included in *T*_0_. The resulting representation of each drug in *T*_0_ is then an *n*−*n*_0_ vector of *EC*_50_ values.

While the representation of each drug will change as the target set *T* changes, the *IC*_50_ values for each of the *m* drugs remains the same. These experimental sensitivity values will be used to test the numerous different target sets to quantify the strength of the model for any target set.

To simplify scoring of the target set, we first convert the *IC*_50_ for each drug *S*_*i*_ to a continuous-valued sensitivity score *y*_*i*_∈ [0,1]

(2) $$\begin{array}{ll} \;y_{i}=\left\{\begin{array}{ll} 1, & \;\text{if}\;IC_{50,i}<\text{Cma}x_{i}\\ c*(1-\text{log}(IC_{50,i})/\text{log}(\text{MaxDos}e_{i}), & \;\text{if}\;\text{Cmax}\leq x\leq \text{MaxDos}e_{i} \end{array}\right) & \; \end{array}$$

where *MaxDose*_*i*_ is the maximum dose of drug *S*_*i*_ given, *Cmax*_*i*_ is the maximum achievable clinical dose of drug *S*_*i*_, and *c*=1−*log*(*Cmax*_*i*_)/*log*(*MaxDose*_*i*_) so that the scoring function is continuous. *MaxDose* is used to prevent inferences being made on data that is not available. While it would be possible to attempt interpolation to infer an *IC*_50_ from the multiple available data points, such inference cannot be fully quantified. Hence, drugs which fail to achieve an *IC*_50_ within the allotted dosage are given the score of 0, which means ineffective. The *Cmax* value is used to apply a variable score to the numerous drugs based on the inherent toxicity of the drug. This will also prevent bias towards drugs with low *IC*_50_’s; some drugs may achieve efficacy at higher levels solely based on the drug *EC*_50_ values.

### Construction of the relevant target set

In this subsection, we present approaches for selection of a smaller relevant set of targets T from the set of all possible targets K. The inputs for the algorithms in this subsection are the binarized drug targets and continuous sensitivity score (output of the algorithms from previous subsection).

With the scaled sensitivities, we can develop a fitness function to evaluate the model strength for an arbitrary set of targets. As has been established, for any set of targets *T*_0_, drug *S*_*i*_ has a unique representation (*S*_*i*_|*T*_0_). This representation can be used to separate the drugs into different bins based on the targets it inhibits under *T*_0_. Within each of these bins will be several drugs with identical target profiles but different scaled scores. Let the set of scores in each bin be denoted *Y*(*S*_*j*_|*T*_0_) for *S*_*j*_ in an arbitrary bin, and we will assign to each bin the mean sensitivity score of the bin, $E(Y(S_{j}|T_{0}))$. Denote this value *P*(*S*_*j*_|*T*_0_). Within each bin, we want to minimize the variation between the predicted sensitivity for the target combination, *P*(*S*_*j*_|*T*_0_), and the experimental sensitivities, *Y*(*S*_*j*_|*T*_0_). This notion is equivalent to minimizing the inconsistencies of the experimental sensitivity values with respect to the predicted sensitivity values for all known target combinations for any set of targets, which in turn suggests the selected target set effectively explains the mechanisms by which the effective drugs are able to kill cancerous cells. Numerically, we can calculate the inter-bin sensitivity error using the following equation:

(3) $$\underset{\text{bins}}{\sum }\underset{j\in \text{bin}}{\sum }|P(S_{j}|T_{0})-Y(S_{j}|T_{0})|$$

This analysis has one notable flaw: if we attempt to only separate the various drugs into bins based on inter-bin sensitivity error, we can create an over-fitted solution by breaking each drug into an individual bin. We take two steps to avoid this. First, we attempt to minimize the number of targets during construction of *T*_0_. Second, we incorporate an inconsistency term to account for target behavior that we consider to be biologically inaccurate.

To expand on the above point, we consider there are two complementary rules by which kinase targets behave. Research has shown that the bulk of viable kinase targets behave as *tumor promoters*, proteins whose presence and lack of inhibition is related to the continued survival and growth of a cancerous tumor. These targets essentially have a positive correlation with cancer progression. This is in opposition to *tumor suppressors*, proteins that have been shown to have a negative correlation with the development of cancer. To capture the behavior of oncogenes, we partially formulate our problem on two rules [34]: 

Rule 1: If (*S*_*i*_|*T*_0_) is the inhibiting set of targets for drug *i* and the drug is successful in inhibiting the circuit, then any set *B* such that *S*_*i*_⊂*B* will also be successful in inhibiting the circuit.

Rule 2: If (*S*_*i*_|*T*_0_) is the inhibiting set of targets for drug *i* and the drug is unsuccessful in inhibiting the circuit, then any set *B* such that *B*⊂*S*_*i*_ will also be unsuccessful in inhibiting the circuit.

Rule 1 essentially says that if inhibiting a number of target proteins has blocked signaling pathways, then inhibiting more target proteins will not open any path that has already been blocked. Rule 2 captures the fact that if a set of target protein inhibitors is unsuccessful in blocking the paths of a circuit, then any reduced number of target protein inhibitors among the inhibiting proteins cannot block all the paths.

The above rules assume that the kinases in focus are oncogenes, genes that promote cancer growth and whose inhibition can prevent tumor development. The majority of kinases in the Drug Screen panel behave as oncogenes, and as such, our approach utilizes the above rules.

Target sets resulting in combination scores that do not follow the rule-based behavior incur an inconsistency penalty. This penalty is calculated as follows:

(4) $$\underset{\text{drugs}}{\sum }\underset{\text{bins}}{\sum }\chi (\text{bin},\text{drug})|P(S_{j}|T_{0})-Y(S_{j}|T_{0})|$$

where *χ*(·) is the indicator function which is 1 when the experimental drug score is inconsistent with the predicted subset/superset bin score.

We now present the complete target set score, and as such, the equation that we wish to solve:

(5) $$\begin{array}{lll} \underset{T}{\min} & \underset{\text{bins}}{\sum }\underset{j\in \text{bin}}{\sum }|P(S_{j}|T)-Y(S_{j}|T)|\; & \;\\ + & \underset{\text{drugs}}{\sum }\underset{\text{bins}}{\sum }\chi (\text{bin},\text{drug})|P(S_{j}|T_{0})-Y(S_{j}|T_{0})|\; \end{array}$$

which reduces to the minimization problem we wish to solve:

(6) $$\begin{array}{lll} \underset{T}{\min}\underset{\text{drugs}}{\sum }\;\underset{\text{bins}}{\sum }\; & (\underset{j\in \text{bin}}{\sum }|P(S_{j}|T)-Y(S_{j}|T)|\; & \;\\ & +\chi (\text{bin},\text{drug})|P(S_{j}|T)-Y(S_{j}|T)|)\; \end{array}$$

For brevity, we will denote the scoring function of a target set with respect to the binarized *EC*_50_ values *S* and the scaled sensitivity scores *Y*, *Γ*(*T*;*S*,*Y*). As the *S* and *Y* sets will be fixed when target set generation begins, we reduce this notation further to *Γ*(*T*).

Note that *T*⊆*K* where *K* denotes the set of all possible targets. 2^|*K*|^ is the total number of possibilities for *T* which is extremely huge and thus prohibits exhaustive search. Thus the inherently nonlinear and computational intensive target set selection optimization will be approached through suboptimal search methodologies. A number of methods can be applied in this scenario and we have employed Sequential Floating Forward Search (SFFS) [35] to build the target sets. We selected SFFS as it generally has fast convergence rates while simultaneously allowing for a large search space within a short runtime. Additionally, it naturally incorporates the desired target set minimization aim as SFFS will not add features that provide no benefit. We present the SFFS algorithm for construction of the minimizing target set in algorithm 1.

#### Algorithm 1 **SFFS Algorithm for generating relevant Target Sets**

#### Complexity of target set generation

The algorithm to generate the error score given a target set *T* is of order $O(m^{2})$, quadratic with respect to the number of drugs. In general, the number of drugs remains relatively low. The SFFS algorithm has a single step runtime of |*K*|, making it linearly increasing with the number of kinase targets. This number is often approximately 300. The total computational cost of selecting a minimizing target set is $O(|K|\cdot m^{2})$. It should be noted this algorithm is extremely parallelizable, and as such adding additional processors allows the effect of the addition of the numerous kinase targets to be computed significantly faster.

### Target combination sensitivity inference from a selected target set

In this subsection, we present algorithms for prediction of drug sensitivities when the binarized targets of the test drugs are provided. The inputs for the algorithms in this subsection are the binarized drug targets, drug sensitivity score and the set of relevant targets for the training drugs.

Construction of the target set that solves Eq. 6 provides information concerning numerically relevant targets based on the drug screen data. However, the resulting model is still limited in its amount of information. Given the binning behavior of the target selection algorithm, the predicted sensitivity values will include only those for which experimental data is provided, and again only a subset of those target combinations. Hence, in order to expand the current model from one of explanation to one that includes prediction, inferential steps have to be applied using the available information.

The first step in inference is prediction of sensitivity values for target combinations outside the known dataset. Consider that the set of drug representations, (*S*|*T*), consists of *c* unique elements. In addition, the number of targets added to the minimizing target set is |*T*|=*n*. The total possible target combinations is then 2^*n*^ for binarized target inhibition, and there are thus 2^*n*^−*c* unknown target combination sensitivities. We would like to be able to perform inference on any of the 2^*n*^−*c* unknown sensitivity combination, and we would like to utilize known sensitivities whenever possible.

To begin the inference step, let us first recall the 2 complementary rules for kinase target behavior upon which we base this model.

**Rule 1**: If (*S*_*i*_|*T*) is the inhibiting set of targets for drug *i* and the drug is successful in inhibiting the circuit, then any set *B* such that *S*_*i*_⊂*B* will also be successful in inhibiting the circuit.

**Rule 2**: If (*S*_*i*_|*T*) is the inhibiting set of targets for drug *i* and the drug is unsuccessful in inhibiting the circuit, then any set *B* such that *B*⊂*S*_*i*_ will also be unsuccessful in inhibiting the circuit.

We consider a third rule that expresses target combination behavior as a function of its most similar target combinations.

**Rule 3**: If (*C*_*i*_|*T*) is the inhibiting set of a target combination with unknown sensitivity, then the sensitivity of (*C*_*i*_|*T*) will be at least that of (*C*_*i*_|*T*)’s closest subsets and will be at most (*C*_*i*_|*T*)’s closest supersets.

Rule 3 follows from the first two rules; rule 1 provides that any superset will have greater sensitivity, and rule 2 provides that any subset will have lower sensitivity.

To apply rule 3 in practical situations, we must guarantee that every combination (*C*_*i*_|*T*) will have a subset and superset with an experimental value. We will assume that the target combination that inhibits all targets in *T* will be very effective, and as such will have sensitivity 1. In addition, the target combination that consists of no inhibition of any target, which is essentially equivalent to no treatment of the disease, will have no effectiveness, and as such will have a sensitivity of 0. Either of these can be substituted with experimental sensitivity values that have the corresponding target combination. In numerous practical scenarios, the target combination of no inhibition has sensitivity 0.

With the lower and upper bound of the target combination sensitivity fixed, we now must perform the inference step by predicting, based on the distance between the subset and superset target combinations. We perform this inference based on binarized inhibition, as the inference here is meant to predict the sensitivity of target combinations with non-specific *EC*_50_ values. Refining sensitivity predictions further based on actual drugs with specified *EC*_50_ values will be considered later.

Let (*C*_*l*_|*T*)= [*k*_1,*l*_,*k*_2,*l*_,⋯,*k*_*n*,*l*_] be the target combination of the subset of (*C*_*i*_|*T*) with the highest sensitivity, and let (*C*_*u*_|*T*)= [*k*_1,*u*_,*k*_2,*u*_,⋯,*k*_*n*,*u*_], the superset target combination with the lowest sensitivity. Let the sensitivity of (*C*_*l*_|*T*) and (*C*_*u*_|*T*) be *y*_*l*_ and *y*_*u*_ respectively. Let the hamming distance between *C*_*l*_ and *C*_*u*_ be *h*=(*C*_*l*_|*T*)⊕(*C*_*u*_|*T*), and the hamming distance between (*C*_*i*_|*T*) and (*C*_*l*_|*T*) be *d*=(*C*_*l*_|*T*)⊕(*C*_*i*_|*T*). Therefore, to transition from (*C*_*l*_|*T*) to (*C*_*i*_|*T*), it will require the inhibition of an additional *d* targets, denoted {*t*_1_,*t*_2_,⋯,*t*_*d*_}, and the remaining *h*−*d*, denoted {*t*_*d*+1_,⋯,*t*_*h*_} targets will remain uncontrolled. For naive inference, we can consider that over the course of the addition of the *h* targets needed to transition from (*C*_*l*_|*T*) to (*C*_*u*_|*T*), the change in sensitivity due to the addition of each target is uniform. With (*C*_*l*_|*T*) as the lower bound of the drug sensitivity, the resulting naive sensitivity from the addition of *d*_2_≤*h* targets is

(7) $$y_{i}(d_{2})=y_{l}+(y_{u}-y_{l})\cdot {\left(\frac{d_{2}}{h}\right)}^{n}$$

where *n* is a tunable inference discount parameter, where decreasing *n* increases *y*_*i*_(*d*_2_) and presents an optimistic estimate of sensitivity.

We can extend the sensitivity inference to a non-naive approach. Suppose for each target *t*_*i*_∈*T*, we have an associated target score *α*_*i*_. The score can be derived from prior knowledge or pre-modeling analysis. Given this vector *α*= [*α*_1_,*α*_2_,⋯,*α*_*n*_], we will define *y*_*i*_ as follows:

(8) $$\begin{array}{lll} y_{i} & (\{t_{1},t_{2},\cdots \;,t_{d}\})=y_{l}+(y_{u}-y_{l})\cdot \; & \;\\ & {\left(\;\frac{\alpha (t_{1})+\alpha (t_{2})+\cdots +\alpha (t_{n})}{\alpha (t_{1})+\alpha (t_{2})+\cdots +\alpha (t_{d})+\alpha (t_{d+1})+\cdots +\alpha (t_{h})}\;\right)}^{n}\; \end{array}$$

which can be written as

(9) $$y_{i}(\{t_{1},t_{2},\cdots \;,t_{d}\})=y_{l}+(y_{u}-y_{l})\cdot {\left(\frac{\overset{d}{\underset{i=1}{\sum }}\alpha (t_{i})}{\overset{h}{\underset{i=1}{\sum }}\alpha (t_{i})}\right)}^{n}$$

As desired, if the majority of the mass of the weights of *t*_1_,*t*_2_,⋯,*t*_*h*_ rest in *t*_1_,*t*_2_,⋯,*t*_*d*_, the sensitivity of *y*_*i*_ will be close to *y*_*u*_.

With the inference function defined as above, we can create a prediction for the sensitivity of any binarized kinase target combination relative to the target set *T*; thus we can infer all of 2^*n*^−*c* unknown sensitivities from the experimental sensitivities, creating a complete map of the sensitivities of all possible kinase target-based therapies relevant for the patient. As noted previously, this complete set of sensitivity combinations constitutes the TIM. The TIM effectively captures the variations of target combination sensitivities across a large target set. However, we also plan to incorporate inference of the underlying nonlinear signaling tumor survival pathway that acts as the underlying cause of tumor progression. We address this using the TIM sensitivity values and the binarized representation of the drugs with respect to target set, (*S*_*i*_|*T*_0_).

### Generation of TIM circuits

In this subsection, we present algorithms for inference of blocks of targets whose inhibition can reduce tumor survival. The resulting combination of blocks can be represented as an abstract tumor survival pathway which will be termed as the TIM circuit. The inputs for this subsection are the inferred TIM from previous subsection and a binarization threshold for sensitivity. The output is a TIM circuit.

Consider that we have generated a target set *T* for a sample cultured from a new patient. With the ability to predict the sensitivity of any target combination, we would like to use the available information to discern the underlying tumor survival network. Due to the nature of the functional data, which is a steady-state snapshot and as such does not incorporate changes over time, we cannot infer models of a dynamic nature. We consider static Boolean relationships. In particular, we expect two types of Boolean relationships: logical AND relationships where an effective treatment consists of inhibiting two or more targets simultaneously, and logical OR relationships where inhibiting one of two or more sets of targets will result in an effective treatment. Here, effectiveness is determined by the desired level of sensitivity before which a treatment will not be considered satisfactory. The two Boolean relationships are reflected in the 2 rules presented previously. By extension, a NOT relationship would capture the behavior of tumor suppressor targets; this behavior is not directly considered in this paper. Another possibility is XOR (exclusive or) and we do not consider it in the current formulation due to the absence of sufficient evidence for existence of such behavior at the kinase target inhibition level.

Thus, our underlying network consists of a Boolean equation with numerous terms. To construct the minimal Boolean equation that describes the underlying network, we utilize the concept of TIM presented in the previous section. Note that generation of the complete TIM would require 2^*n*^−*c*≈2^*n*^ inferences. The inferences are of negligible computation cost, but for a reasonable *n*, the number of necessary inferences can become prohibitive as the TIM is exponential in size. We assume that generating the complete TIM is computationally infeasible within the desired time frame to develop treatment strategies for new patients. Thus, we fix a maximum size for the number of targets in each target combination to limit the number of required inference steps. Let this maximum number of targets considered be M.

We then consider all non-experimental sensitivity combinations with fewer than *M*+1 targets. As we want to generate a Boolean equation, we have to binarize the resulting inferred sensitivities to test whether or not a target combination is effective. We denote the binarization threshold for inferred sensitivity values by *θ*∈ [0,1]. As *θ*_*i*_→1, an effective combination becomes more restrictive, and the resulting boolean equations will have fewer effective terms. There is an equivalent term for target combinations with experimental sensitivity, denoted *θ*_*e*_.

We begin with the target combinations with experimental sensitivities. For converting the target combinations with experimental sensitivity, we binarize those target combinations, regardless of the number of targets, where the sensitivity is greater than *θ*_*e*_. The terms that represent a successful treatment are added to the Boolean equation. Furthermore, the terms that have sufficient sensitivity can be verified against the drug representation data to reduce the error. To find the terms of the network Boolean equation, we begin with all possible target combinations of size 1. If the sensitivity of these single targets are sufficient relative to *θ*_*i*_ and *θ*_*e*_, the target is binarized; any further addition of targets will only improve the sensitivity as per rule 3. Thus, we can consider this target completed with respect to the equation, as we have created the minimal term in the equation for the target. If the target is not binarized at that level, we expand it by including all possible combinations of two targets including the target in focus. We continue expanding this method, cutting search threads once the binarization threshold has been reached.

#### Algorithm 2 **Algorithm for generation of minimal boolean equation**

The method essentially resembles a breadth or depth-first search routine over *n* branches to a maximum depth of *M*. This routine has time complexity of *O*(*D*∗*n*^*M*^), and will select the minimal terms in the Boolean equation. The *D* term results from the cost of a single inference. The time complexity of this method is significantly lower than generation of the complete TIM and optimizing the resulting TIM to a minimal Boolean equation. For the minimal Boolean equation generation algorithm shown in algorithm 2, let the function *binary*(*x*|*T*) return the binary equivalent of *x* given the number of targets in *T*, and let *sensitivity*(*x*|*T*) return the sensitivity of the inhibition combination *x* for the target set *T*.

With the minimal Boolean equation created using Algorithm 2, the terms can be appropriately grouped to generate an equivalent and more appealing minimal equation. To convey the minimal Boolean equation to clinicians and researchers unfamiliar with Boolean equations, we utilize a convenient circuit representation, as in Figures 2 and 3. These circuits were generated from two canine subjects with osteosarcoma, as discussed in the results section.

The circuit diagrams are organized by grouped terms, which we denote as *blocks*. Blocks in the TIM circuit act as possible treatment combinations. The blocks are organized in a linear OR structure; treatment of any one block should result in high sensitivity. As such, inhibition of each target results in its line being broken. When there are no available paths between the beginning and end of the circuit, the treatment is considered effective. As such, each block is essentially a modified AND/OR structure. Within the blocks, parallel lines denote an AND relationship, and adjacent lines represent an OR relationship. The goal of an effective treatment then, from the perspective of the network circuit diagram, is to prevent the tumor from having a pathway by which it can continue to grow.

## Discussion

In this section, we discuss extensions of the TIM framework presented earlier. We provide foundational work for incorporating sensitivity prediction via continuous-valued analysis of *EC*_50_ values of new drugs as well as theoretical work concerning dynamical models generated from the steady state TIMs developed previously.

### Incorporating continuous target inhibition values

The analysis considered in the earlier sections was based on discretized target inhibition i.e. each drug was denoted by a binary vector (*S*|*T*) representing the targets inhibited by the drug. The framework can predict the sensitivities of new drugs with high accuracy as illustrated by the results on canine osteosarcoma tumor cultures. However, the current framework can also be modified to incorporate the continuous nature of target inhibition and application of different concentrations of a new drug. Let us consider that a drug *i* with target set *T*_0_ and *EC*_50_ profile *e*_*i*,1_,*e*_*i*,2_,⋯,*e*_*i*,*n*_ is applied at concentration *x* nM. For each *EC*_50_ value *e*_*i*,*j*_, we can fit a hill curve or a logistic function to estimate the inhibition of target *j* at concentration *x* nM. For instance a logistic function will estimate the inhibition of target *j* as $f(j|x)=1/(1+e^{\text{log}(e_{i,j}/x)})$. Note that at concentration *x*=*e*_*i*,*j*_, *f*(*j*|*x*)=0.5 as desired. This approach can be applied to arrive at a continuous target profile *z*_*i*,1_,*z*_*i*,2_,⋯,*z*_*i*,*n*_ of a drug that is dependent on the applied drug concentration. The *z*_*i*,*j*_’s denote real numbers between 0 and 1 representing the inhibition ratio of target *j*. This approach can also be applied to generate drug target profiles for a combination of drugs at different concentrations. To arrive at the sensitivity prediction for a new target inhibition profile, we can apply rules similar to Rules 1, 2 and 3 along with searching for closest target inhibition profiles among the training data set. The block analysis performed using discretized target inhibitions can provide smaller sub-networks to search for among the target inhibition profiles.

#### Incorporating network dynamics in the TIM formulation

**Figure 4:**
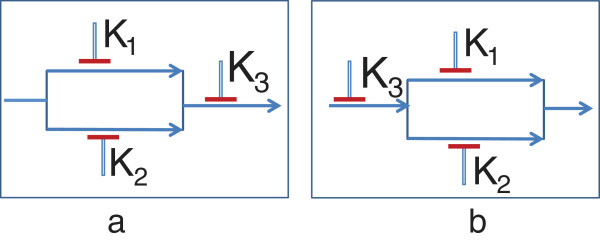
**Feasible dynamical model structures.** Two possible dynamical models of a 3 target system **(a)** K1 and K2 are activated due to mutations which in turn activates K3 **(b)** K3 is activated due to mutation which in turns activate K1 and K2.

The TIM developed in the previous sections is able to predict the steady state behavior of target inhibitor combinations but cannot provide us with the dynamics of the model or the directionality (upstream or downstream) of the tumor pathways. This limitation is a result of the experimental drug perturbation data being from the steady state. Our results show that the proposed approach is highly successful in locating the primary faults in a tumor circuit and predict the possible sensitivity of target combinations at the current time point. However, extension of this model to incorporate the directional pathways will require protein or gene expression measurements. The extension refers to steps F1 and F2 in Figure 1. These steps are not necessary to design the control policy but if performed can provide superior performance guarantees. If we plan to infer a dynamic model from no prior knowledge, the number of required experiments will be huge and will primarily require time- series gene or protein expression measurements. In this section, we will show that the circuit produced by our TIM approach can be used to significantly reduce the search space of directional pathways. To arrive at the potential dynamical models satisfying the inferred TIM, we will consider the possible directional pathways that can generate the inferred TIM and convert the directional pathways to discrete Boolean Network (BN) [36] models. The TIM can be used to locate the feasible mutation patterns and constrain the search space of the dynamic models generating the TIM. For the duration of the Network Dynamics analysis, we will consider the two dynamic models shown in Figure 4.

##### Directional pathway to BN

To generate a discrete dynamical Boolean Network (BN) model [36] of a directional pathway, we will first consider the starting mutations or latent activations. The number of states in the BN will be 2^*n*+1^ for *n* targets. Each state will have *n*+1 bits with first *n* bits referring to the discrete state of the *n* targets and the least significant bit (LSB) will correspond to the binarized phenotype ie. tumor (1) or normal (0). The rules of state transition are (a) A target state at time *t*+1 becomes 1 if any immediate upstream neighbor has state 1 at time *t* for *OR* relationships or all immediate upstream neighbors have state 1 at time *t* for *AND* relationships. Note that the examples have *OR* type of relations as they are the most commonly found relations in biological pathways (based on illustrated pathways in KEGG). (b) For the BN without any drug, the targets that are mutated or have latent activations will transition to state 1 within one time step. (c) For a target with no inherent mutation or latent activation, the state will become 0 at time *t*+1 if the immediate upstream activators of the target has state 0 at time *t*.

**Figure 5:**
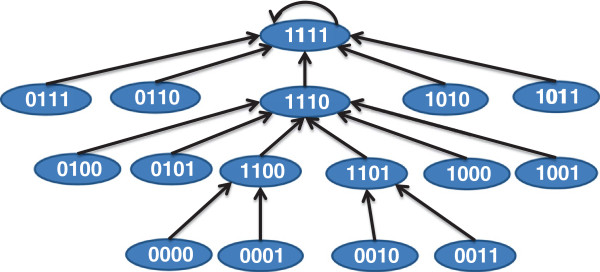
**State transitions of the BN for the directional pathway in Figure **4**(a).**

Let us consider the simple example of a biological pathway shown in Figure4(a). The downstream target *K*_3_ can be activated by either of the upstream targets *K*_1_ or *K*_2_. The tumor is in turn caused by the activation of *K*_3_. For this directional pathway, we will assume that *K*_1_ and *K*_2_ are activated by their own mutations or have latent activations. The corresponding BN transition diagram for this pathway is shown in Figure 5. For instance, if we consider the state 0010 at time *t*, it denotes *K*_1_, *K*_2_ being inactive and *K*_3_ being active and the phenotype being non-tumorous. Based on the directional pathway in Figure 4(a), activation of *K*_3_ causes tumor and thus the phenotype will change to tumor (*i.e.* 1) at *t*+1. We are given that only *K*_1_ and *K*_2_ have mutations or latent activations, thus the activation *K*_3_ cannot be maintained without the activation of either *K*_1_ or *K*_2_ and thus we will have *K*_3_=0 at *t*+1. However, since *K*_1_ and *K*_2_ have mutations or latent activations, they will become 1 at time *t*+1 which in turn will activate *K*_3_ at time *t*+2.

##### Dynamical model following target inhibition

The BN in Figure 5 can also be represented by a 16×16 transition matrix *Q* representing the state transitions. To generate the dynamic model after inhibition of a specific target set *S*_1_ (by application of targeted drugs), we should consider that the transition *i*→*j* in the un-treated system will be converted to *i*→*z* in the treated system where *z* differs from *j* only in the target set *S*_1_ and all targets in *S*_1_ have value 0 for *z*. Each target inhibition combination can be considered as multiplying a matrix *T*_*c*_ to the initial transition matrix *Q*. Each row of *T*_*c*_ contains only one non-zero element of 1 based on how the inhibition alters the state. If we consider *n* targets, *nT*_*c*_’s in combination can produce a total of 2^*n*^ possible transformation matrices $T_{1},T_{2},\cdots \;,T_{2^{n}}$. The TIM denotes the state of the LSB of the attractor for the 2^*n*^ transition matrices $T_{1}Q,T_{2}Q,\cdots \;,T_{2^{n}}Q$ starting from initial state 11⋯1 (*i.e.* all targets considered in the TIM and tumor are activated).

**Figure 6:**
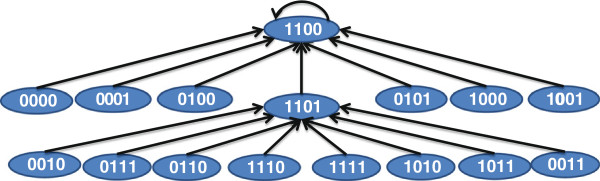
**BN state transition following inhibition of target*K***_**3**_**.**

**Figure 7:**
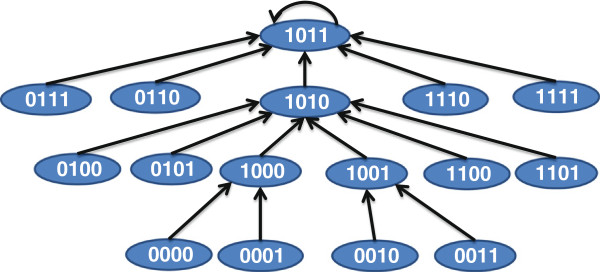
**BN state transitions following inhibition of target*K***_**2**_**.**

For instance, if we consider that our drug inhibits the target *K*_3_ (*i.e.* set *S*_1_={*K*_3_}), the discrete dynamic model following application of the drug is shown in Figure 6. We should note that the equilibrium state of the network 1100 has 0 for the tumor state. This is because the tumor is activated by *K*_3_ and inhibition of *K*_3_ should eradicate the tumor. On the other hand, since both *K*_1_ and *K*_2_ can cause tumor through activation of intermediate *K*_3_, inhibition of only one of *K*_1_ and *K*_2_ will not block the tumor. The BN following inhibition of *K*_2_ is shown in Figure 7 where the attractor 1011 denotes a tumorous phenotype.

##### Experiment design to infer the dynamic pathway structure

The TIM can be used to produce possible dynamic models based on assumptions of latent activations or mutations. For instance, knowledge of the steady-state value of the target *K*_1_ following application of target inhibitor for *K*_3_, will remove one of the possibilities. Following inhibition of *K*_3_, the value of *K*_1_ will remain 1 for the case of Figure 4(a) as *K*_1_ is upstream of *K*_3_. Conversely, the value of *K*_1_ will be 0 for the second case as *K*_3_ activates *K*_1_.

**Figure 8:**
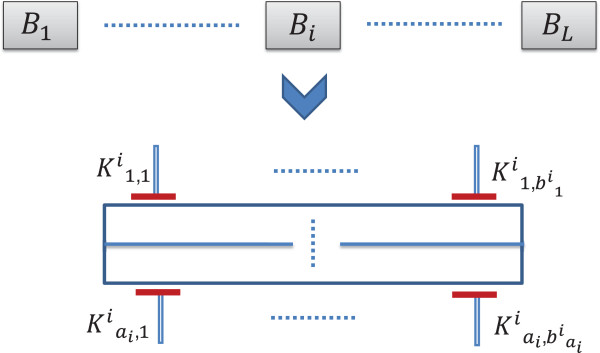
A general abstract pathway resulting from a target inhibition map.

In the following paragraphs, we will consider a general pathway obtained from a TIM having the structure shown in Figure 8 but with unknown directionalities of the blocks and target positions. For the current analysis, we will assume that there are no common targets in distinct blocks. We will consider that the pathway has *L* blocks in series (*B*_1_,*B*_2_,⋯,*B*_*L*_) and each block *B*_*i*_ has *a*_*i*_ parallel lines with each line *j* containing $b_{j}^{i}$ targets ($K_{1,1}^{i},K_{1,2}^{i}\cdots \;,K_{1,b_{j}^{i}}^{i}$). The total number of targets in the general map is $\overset{L}{\underset{i=1}{\sum }}\overset{a_{i}}{\underset{j=1}{\sum }}b_{j}^{i}$.

Assuming that the *n* targets are distinct, the maximum number of distinct discrete dynamic models satisfying the structure is $L!\overset{L}{\underset{i=1}{\prod }}\overset{a_{i}}{\underset{j=1}{\prod }}(b_{j}^{i})!$. Each parallel line *j* in block *i* can have *bji*! possible directional orientations.

If the Figure 8 represents a possible directional orientation, then only the targets $K_{1,1}^{1},K_{2,1}^{1}\cdots K_{a_{1},1}^{1}$ will have initial activations due to mutations or latent activations. Some other downstream target cannot have a mutation or latent activation otherwise the target inhibition combination $K_{1,1}^{1},K_{2,1}^{1}\cdots K_{a_{1},1}^{1}$ will not be effective.

For our analysis, we are assuming that we can inhibit specific targets of our choice and we can measure the steady state target expression following application of the target inhibitions.

We can locate the directionality of the blocks *B*_1_ to *B*_*L*_ by using at most *L*−1 steady state measurements. We can start by randomly picking any block *B*_*i*_ and blocking the targets in that block, the blocks that will remain activated will be upstream of that block and the blocks that will be deactivated following the inhibition of block *B*_*i*_ will be located down-stream of *B*_*i*_.

Note that the number of experiments required is based on steady state measurements following particular perturbations. Time series measurements can reduce the number of experiments required but may not be always technically feasible.

The expected number of experiments required to detect the directionality of *L* serial blocks is

(10) $$\frac{2L-1}{3}\text{for}\;L\geq 2$$

The next step will be locating the directionality of targets in each parallel line of the block. We can start with an experiment where for each block *B*_*i*_, one target from each line up to a maximum of *a*_*i*_−1 lines will be inhibited. We cannot inhibit all the lines in a block or else the downstream blocks will also be inhibited and no inference can be made on those blocks for that experiment. While locating the directionality of the serial blocks *B*_*i*_, we have already validated the position of one target from each parallel line in a serial block.

If we consider a single block *B*_*i*_, each experiment can detect the location of *a*_*i*_−1 targets, thus the total number of experiments required to decipher the possible directionalities of the targets in the block *B*_*i*_ is $\leq \max(\underset{j\in S_{i}}{\max}\overset{i}{\underset{j}{b}}-2,\lceil \frac{\underset{j\in S_{i}}{\sum }\overset{i}{\underset{j}{b}}-a_{i}}{a_{i}-1}\rceil -1)$ where *S*_*i*_={1,⋯,*a*_*i*_}.

Thus for the overall map, the worst case number of experiments $N_{E}^{w}$ required to decipher the directionalities of all the targets is upper-bounded by

(11) $$\begin{array}{lll} & N_{E}^{w}\leq \underset{i\in S}{\max}\{\max(\underset{j\in S_{i}}{\max}\overset{i}{\underset{j}{b}}-2,\; & \;\\ & \;\lceil \frac{\underset{j\in S_{i}}{\sum }b_{j}^{i}-a_{i}}{a_{i}-1}\rceil -˘\;1)\}+L-1\; \end{array}$$

where *S*={1,⋯,*L*}. Utilizing equation 10, the expected number of experiments $N_{E}^{a}$ required to decipher the directionalities of all the targets is upper-bounded by

(12) $$\begin{array}{lll} & N_{E}^{a}\leq \underset{i\in S}{\max}\{\max(\underset{j\in S_{i}}{\max}\frac{2\overset{i}{\underset{j}{b}}-4}{3},\; & \;\\ & \;\lceil \frac{\underset{j\in S_{i}}{\sum }2b_{j}^{i}-a_{i}}{3(a_{i}-1)}\rceil -˘\;1)\}+\frac{2L-1}{3}\; \end{array}$$

## Conclusions

In this article, we presented a novel framework for predicting the effectiveness of molecularly targeted drugs. We used drug perturbation data to generate a map of the underlying genetic regulatory pathway. Using actual experimental data, we were able to show the effectiveness of our approach for drug sensitivity prediction. The proposed TIM approach produced a low average leave one out cross validation error of 5% when applied to perturbation data generated from four primary canine tumors using a set of 60 drugs. We should note that the current 60 drug screen is a small one and technology has been developed for drug screens with a far greater number of drugs. We are currently experimenting with pharmaceutical drug library consisting of more than 300 small molecule inhibitors. We expect that the use of larger number of drugs will increase the accuracy further and generate maps with greater robustness. The scope of the present article is concentrated around steps B, C and D of Figure 1.

For future research, we will consider multiple data sources to increase the robustness of the designed maps. As explained in Figure 1, we can use RAPID siRNA screens to validate single points of failures predicted by our TIM approach. Furthermore, RNAseq and protein phosphoarray data can be used to further revise the circuit. Finally, time series data can be used to incorporate dynamics in the modeling framework. For combination therapy design, we can use the TIM framework to formulate control strategies with various constraints. Some possibilities are (a) minimal toxicity, (b) anticipating evolving drug resistance, and (c) success over a family of TIMs representing variations of a tumor. For case (a), we can assume that the toxicity of a drug or drug combination is proportional to the number of targets being inhibited by the drug(s) and search for the drug combination with high sensitivity but low set of target inhibitions. For case (b), we would want to avoid resistance and thus would like to inhibit more than one independent blocking pathway such that for the scenario when resistance to one of the blocking pathways develops, the other independent pathway(s) can still keep the tumor under check. In other words, we would be interested in selecting a set of targets that can be divided into two or more non-intersecting sets such that the sensitivity of each set is higher than a threshold. For case (c), the goal is to design control policies for the scenario when the exact pathway is not known but it belongs to a collection of pathways. The uncertainty can arise when the experimental data is not sufficient enough to produce a unique pathway map or the current pathway may evolve into one of the different pathways obtained from tissues with same type of cancer. This can approached from a worst case perspective [37] or a Bayesian perspective [38].

In conclusion, the proposed framework provides a unique input-output based methodology to model a cancer pathway and predict the effectiveness of targeted drugs. This framework can be developed as a viable approach for personalized cancer therapy. To aide in the usage of our framework, we have developed a Graphical User Interface which implements in an easy to use way the algorithms and equations presented in this paper. It is built in MATLAB, but is distributed as a compiled executable; as such, it is usable in a Windows environment by downloading the MATLAB Compile Runtime (MCR) Environment, which is free to download and requires no MATLAB installation. It is available online at: http://cvial.ece.ttu.edu/ranadippal/research.html under the *Target Inhibition Map approach to inference of cancer pathways* heading.

## Competing interests

Author CK has received honoraria for scientific presentations at Novartis, Millennium/Takeda Pharmaceutical and GlaxoSmithKline, and has research joint ventures or sponsored research with Novartis and Johnson & Johnson. CK is also a paid consultant to the NCI/CTEP Pediatric Preclinical Testing Program (PPTP). CK is also a co-founder/stockholder of Numira Biosciences. The remaining authors have no other conflicts to declare related to these studies except that a EC is a first degree relative of a BMS scientist.

## Authors’ contributions

Designed the algorithmic framework: NB, RP. Implemented the algorithms: NB. Performed the experiments: LD, EC, BS, CK. Analyzed the results: NB, RP. All authors read and approved the final manuscript.

## Supplementary Material

Additional file 1 **Supplementary Analysis.** The file includes a detailed TIM example, results of a set of synthetic experiments, theoretical analysis of errors and additional TIM circuits. Click here for file

Additional file 2 **Drug Screen*IC***_***50***_** dataset.** The experimentally measured *IC*_50_’s in nM for four cell cultures (Bailey, Charley, Sy, Cora) across 60 drugs. Click here for file

Additional file 3 **Drug Screen Panel*EC***_***50***_**.** The *EC*_50_ values in nM for 404 targets for 46 drugs. Click here for file

Additional file 4 **Cross validation results.** The detailed results of the 10 fold cross validation error estimates for the tumor cultures Bailey, Charley, Sy and Cora. Click here for file

Additional file 5 **Similarity between Drugs.** The similarities between the 44 targeted drugs as measured by similarity measure *Δ*. Click here for file
