# Supplementary material for: A new approach for prediction of tumor sensitivity to targeted drugs based on functional data
Source: BMC Bioinformatics. 2013 Jul 29;14:239. doi: 10.1186/1471-2105-14-239 (PMC3750584; doi:10.1186/1471-2105-14-239)
Supplement: Additional file 1 — Supplementary Analysis. The file includes a detailed TIM example, results of a set of synthetic experiments, theoretical analysis of errors and additional TIM circuits. [file 1471-2105-14-239-S1.pdf]

Supplementary Analysis

1 TIM Example

To explain the Target Inhibition Map (TIM), let us consider a simple example of a pathway as shown in figure 4(a). The downstream target K_3 can be activated by either of the upstream targets K_1 or K_2. The tumor is in turn caused by the activation of K_3. For this directional pathway, we will assume that K_1 and K_2 are activated by their own mutations or have latent activations. We note that the tumor proliferation (tumor activation) can be stopped by inhibiting K_3 or inhibiting both of K_1 and K_2. We can consider this as two series blocks (Block1= $\{K_1, K_2\}$ and Block2 = $\{K_3\}$) that independently can reduce tumor proliferation. Suppose Block1 can reduce tumor proliferation by 80% and Block 2 can reduce tumor proliferation by 70% , then we will consider that inhibition of both blocks simultaneously will reduce tumor proliferation by (1-(1-0.8)(1-0.7))*100= 94%. Note that we consider the sensitivity to be 1 - the ratio of tumor cells remaining after drug application as compared to without drug application. Thus 94% reduction of tumor cells produces a sensitivity of 0.94. Thus, if we consider the sensitivities for different combinations of target inhibitions, we will arrive at a truth table of the sort shown in table A.1. The continuous sensitivities have been binarized using a threshold of 0.5. Note that, it is not required to test all the possible $2^3 = 8$ target combinations for arriving at the column of sensitivities. For instance, if we consider the binarized sensitivities and our experimental data shows that inhibition of $K_1, K_2, K_3 = \{0, 0, 1\}$ produces a sensitivity of 1, then we know that $K_1, K_2, K_3 = \{1, 0, 1\}; \{0, 1, 1\}; \{1, 1, 1\}$ will also have a sensitivity of 1. Thus, in this particular case, we can estimate the sensitivities of 4 possible target combinations based on a single experiment.

Table A.1: Target inhibitions and corresponding sensitivities for pathway in figure 4(a)

Target Inhibition			Sensitivity (continuous)	Sensitivity (binarized)	Tumor Survival
K_1	K_2	K_3	S	$B(S)$	$1 - B(S)$
0	0	0	0	0	1
0	0	1	0.7	1	0
0	1	0	0	0	1
0	1	1	0.7	1	0
1	0	0	0	0	1
1	0	1	0.7	1	0
1	1	0	0.8	1	0
1	1	1	0.94	1	0

We next provide another set of synthetic simulation experiments to estimate the accuracy of the prediction

algorithm.

2 Synthetic Experiment 2

We consider that the biological tumor proliferation pathways consists of multiple blocks as shown in figures 2, 3 and 8. The tumor proliferation can be reduced by inhibiting all the targets in a block. For the simulation, cases like the block EHMT1 and CDK4, AKT1 in figure 3 is considered as two blocks consisting of {EHMT1, CDK4} and {EHMT1, AKT1}. The number of blocks for each random synthetic pathway is selected to be a random number between NB_{min} and NB_{max}. The number of targets in a block is a random number between 1 and $MaxBlockSize$. The sensitivity of each block is selected to be a random number between 0 and 1 that satisfies the biological constraints of Rule 1 and Rule 2. The final sensitivity when multiple blocks are inhibited is calculated similar to the manner described in the preliminary example. For T number of targets, there can be 2^T different drugs and once a synthetic pathway is created, a set of N random dugs from the possible 2^T drugs is selected to create the training samples. The prediction results are then tested on a separate M drugs selected random from the 2^T drugs. As an example, if $T = 12$, there can be $2^{12} = 4096$ possible drugs and we tested with 100 training drugs which constitute less than 2.5 percent of possible drugs and the sensitivities of the remaining 97.5 percent drugs are unknown.

Table B.2: Simulation Results for Synthetic Pathway Experiment 2. Here MB=$MaxBlockSize$, N1=NB_{Min}, N2= NB_{Max}, NT=Number of Targets, NTR=Number of Training Samples, NTS=No. of Test Samples, NP=Noumber of Different pathways, MAE = Mean Absolute Error, MC = Mean Correlation Coefficient, ESD= Mean Error Standard Deviation

						Proposed Algorithm			Random Predictions		
MB	[N1 N2]	NT	NTR	NTS	NP	MAE	MC	ESD	MAE	MC	ESD
4	[5 10]	12	100	100	25	0.093	0.91	0.17	0.44	0.02	0.51
5	[5 10]	12	200	400	25	0.07	0.93	0.15	0.44	-0.01	0.51
4	[5 10]	10	200	400	25	0.046	0.96	0.12	0.44	0.01	0.51
4	[5 10]	10	100	100	25	0.071	0.93	0.14	0.45	-0.03	0.52
5	[5 15]	15	250	750	25	0.09	0.92	0.17	0.44	0	0.52
5	[5 15]	15	100	200	25	0.11	0.88	0.19	0.45	0	0.51
4	[5 15]	20	200	300	10	0.115	0.88	0.19	0.44	0.02	0.51
4	[5 15]	20	500	500	10	0.091	0.91	0.17	0.43	0.02	0.5

The simulation results are shown in Table B.2. In table B.2, MB refers to the maximum number of targets in a single block, the number of blocks for each synthetic pathway is between N1 and N2, NT provides the number of targets, NTR and NTS provides the number of training and testing samples for each synthetic pathway, NP provides the number of pathways generated. For these simulation parameters, the

predictions results of our proposed algorithm is provided as the mean absolute error (MAE) which denotes the expectation over the pathways of the average absolute error of each pathway for the testing samples. The MC denotes the average correlation coefficient between the actual synthetic pathway sensitivities and predicted sensitivities for the testing samples. The ESD denotes the mean standard deviation of the error in prediction for the testing samples. The next three columns denotes the MAE, MC and ESD if we predict the sensitivities using uniform random numbers between 0 and 1. The first row shows that for 12 targets and 100 training samples, we are able to achieve a MAE of 0.093 and a MC of 0.91 while random predictions have a MAE of 0.44 and MC of 0.02. For our synthetic pathway simulation experiments, the correlation coefficient between predicted and actual sensitivity is close to 0.9 and the MAE is around 0.1 showing the high prediction accuracy of our proposed algorithm.

Figure B.1 shows the histogram of the errors in prediction for the set of synthetic pathway results shown in second row of table B.2. Note that with NTS=400 and NP=25, there were a total of 400×25=10,000 prediction errors. The histogram in B.1 shows that a large number of sample errors are close to 0 with 10 percentile being -0.154 and 90 percentile being 0.051.

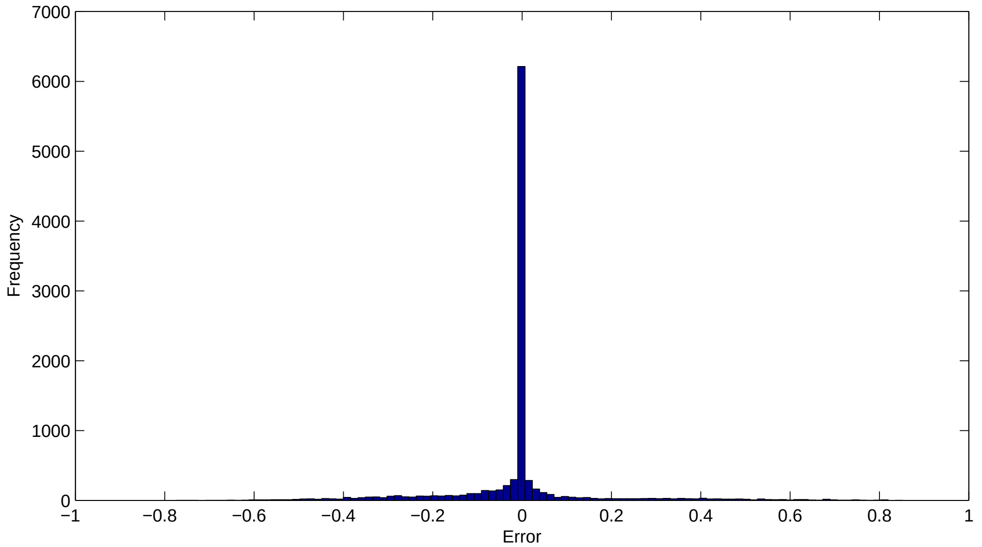

Figure B.1: Histogram of errors for $400 \times 25 = 10000$ testing sample predictions for case 2 in Table B.2.

3 Prediction Error Analysis

In this section, we analyze the prediction error resulting from each serial pathway block. Let us consider that a block has β targets. Usually the β will be ≤ 6. Let us consider that the training set consists of η samples that are independently selected based on the probability distribution $f_{X_1,X_2,\cdots,X_\beta}$. We are interested in predicting the block sensitivity of a new sample with the following target inhibition profile $V = y_1, y_2, \cdots, y_\beta$ where $y_i = 0$ or 1 for $i \in \{1, 2, \cdots, \beta\}$. Let the number of 1's in V be denoted by ω and 0's by $\beta - \omega$. Let A denote the event that a sample $x_1, x_2, \cdots, x_\beta$ selected based on the probability distribution $f_{X_1,X_2,\cdots,X_\beta}$ has the relation $(x_1 \leq y_1) \wedge (x_2 \leq y_2) \wedge \cdots (x_\beta \leq y_\beta)$.

Then assuming $X_1, \ldots X_\beta$ are identically and independently distributed with equal probability of selecting 1 or 0, we have

$$P(A) = \frac{1}{2^{\beta-\omega}} \tag{12}$$

Similarly, let B denote the event that a sample $x_1, x_2, \cdots, x_\beta$ selected based on the probability distribution $f_{X_1,X_2,\cdots,X_\beta}$ has the relation $(x_1 \geq y_1) \wedge (x_2 \geq y_2) \wedge \cdots (x_\beta \geq y_\beta)$ and at least one $x_i > y_i$ for $i = 1, 2, \cdots, \beta$.

Thus,

$$P(B) = \frac{2^{\beta-\omega} - 1}{2^\omega} = \frac{1}{2^\omega} - \frac{1}{2^\beta} \tag{13}$$

The number of samples out of the training set that satisfies events A and B follows binomial distributions $Binomial(\eta, P(A))$ and $Binomial(\eta, P(B))$ respectively. Thus, the expected number of samples for events A and B are $\eta * P(A)$ and $\eta * P(B)$. As a numerical example, if number of samples $\eta = 60$ and block size $\beta = 6$ and for equal number of 1's $\omega = 3$, then the expected number of samples for event A= 7.5 and for event B = 6.56. For this example, the number of samples in combined events A and B will always be greater than 14 for all ω's.

Next, let us estimate the error in prediction when we estimate based on maximum sensitivity among points in A and minimum sensitivity among points in B. We will consider that A and B contain n_1 and n_2 points respectively with $n_1 + n_2 = \lambda$. Let the sensitivities of the λ points be distributed uniformly in [0 1]. We will also add two more points $0, 0, \cdots, 0$ and $1, 1, \cdots, 1$ with sensitivities 0 and 1 respectively. Let the sorted $\lambda + 2$ sensitivities be $0 \leq s_1 \leq s_2 \cdots \leq s_\lambda \leq 1$. Let us denote the maximum sensitivity among the n_1 points in A by Y_l and the minimum sensitivity among the n_2 points in B by Y_h. Based on biological constraints, the actual sensitivity for $y_1, y_2, \cdots, y_\beta$ lies between Y_l and Y_h. Without any other information, we will consider that the actual sensitivity Y_{ac} follows a uniform distribution $f_{Y_{ac}}$ between Y_l and Y_h. Thus

if we consider a basic prediction of $Y_p = (Y_l + Y_h)/2$ for our unknown sensitivity, the expected error in prediction for given Y_l and Y_h is $\mathbb{E}_{Y_{ac}}(|Y_{ac} - Y_p| \ \ |Y_l, Y_h, \lambda) = \int_{Y_l}^{Y_h} |x - Y_p| f_{Y_{ac}}(x)dx$. For uniform sensitivity in the range $[Y_l \ \ Y_h]$, the expected error is $(Y_h - Y_l)/4$. Thus,

$$\begin{aligned}\mathbb{E}_{Y_l,Y_h,Y_{ac}}(|Y_{ac} - Y_p| \ \ |\lambda) &= \mathbb{E}_{Y_l,Y_h}(\mathbb{E}_{Y_{ac}}(|Y_{ac} - Y_p| \ \ |Y_l, Y_h, \lambda)) \\ &= \frac{\mathbb{E}(Y_h) - \mathbb{E}(Y_l)}{4} \qquad (14)\end{aligned}$$

For the sorted $\lambda + 2$ sensitivities $0 \leq s_1 \leq s_2 \cdots \leq s_\lambda \leq 1$, $\mathbb{E}(s_i) - \mathbb{E}(s_{i-1}) = 1/(\lambda+1)$. Thus the expected error for a given λ is $\mathbb{E}(|Y_{ac} - Y_p| \ \ |\lambda) = \frac{1}{4*(\lambda+1)}$. To calculate the expected error based on the possibilities of λ, we note that λ follows a binomial distribution $Binomial(\eta, P(A) + P(B))$. Thus the expected error is

$$\begin{aligned}\mathbb{E}(|Y_{ac} - Y_p|) &= \sum_{\lambda=0}^{\eta} \frac{1}{4*(1+\lambda)} \binom{\eta}{\lambda} (P(A) + P(B))^\lambda (1 - P(A) - P(B))^{\eta-\lambda} \\ &= \frac{1 - (1 - P(A) - P(B))^{\eta+1}}{4*(\eta+1)*(P(A)+P(B))} \qquad (15)\end{aligned}$$

We also have $P(A) + P(B) = \frac{2^\omega + 2^{\beta-\omega} - 1}{2^\beta} \geq \frac{1}{2^{\beta/2-1}} - \frac{1}{2^\beta}$.

As a numerical example, when $P(A) + P(B) = \frac{1}{2^{\beta/2-1}} - \frac{1}{2^\beta}$ and $\eta = 100$, then $\mathbb{E}(|Y_{ac} - Y_p|) = 0.0057, 0.0106, 0.0204$ for $\beta = 4, 6, 8$ respectively. Thus we note that the sensitivities for each individual block can be predicted with high precision.

4 TIM Circuits

The inferred circuits for primary cultures Charley and Cora are shown in Figs D.2 and D.3 respectively.

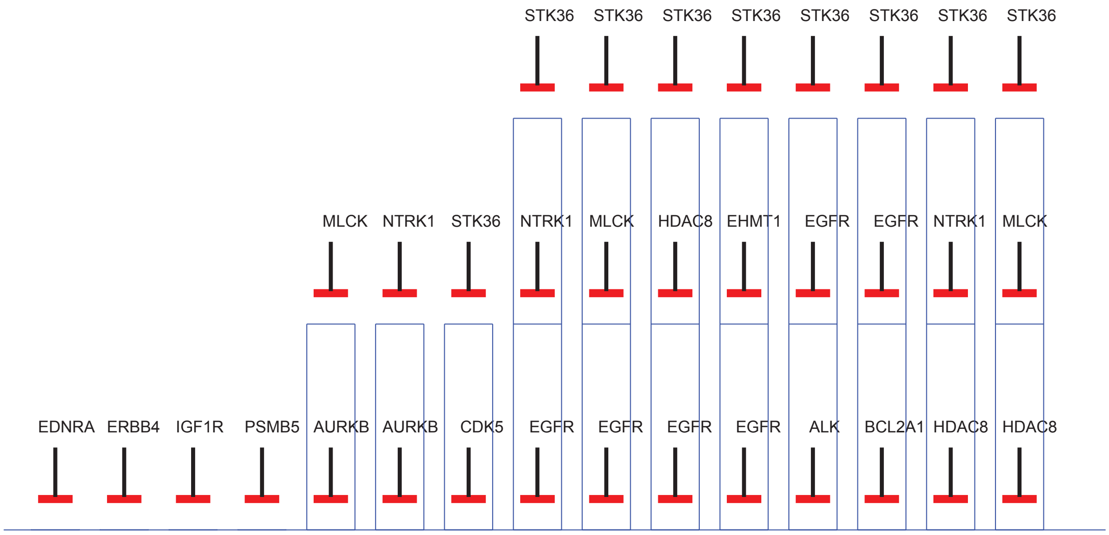

Figure D.2: TIM Circuit for Osteosarcoma Primary Culture Charley

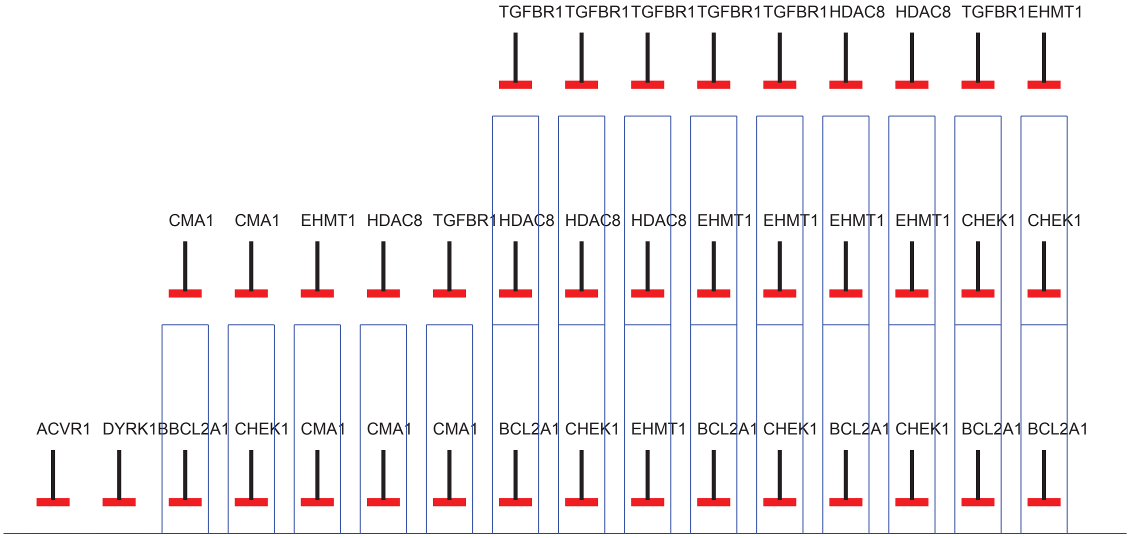

Figure D.3: TIM Circuit for Osteosarcoma Primary Culture Cora
